# Preparation of Poloxamer188-*b*-PCL and Study on *in vitro* Radioprotection Activity of Curcumin-Loaded Nanoparticles

**DOI:** 10.3389/fchem.2020.00212

**Published:** 2020-04-15

**Authors:** Xiaona Lin, Yongli Shi, ShaSha Yu, Siyi Li, Wenhui Li, Meishuang Li, Shengxi Chen, Yuanbo Wang, Mei Cong

**Affiliations:** ^1^Tianjin Key Laboratory of Radiation Medicine and Molecular Nuclear Medicine Institute of Radiation Medicine, Chinese Academy of Medical Sciences and Peking Union Medical College, Tianjin, China; ^2^College of Pharmacy, Xinxiang Medical University, Xinxiang, China

**Keywords:** poloxamer188-*b*-PCL, nanoparticles, antioxidant activity, curcumin, caner radiotherapy

## Abstract

A novel polymer of poloxamer188-*b*-PCL was synthesized via a ring-opening polymerization. Fourier transform infrared spectroscopy (FTIR), Raman, and ^1^H nuclear magnetic resonance (^1^H NMR) spectra were used to study the structures of obtained poloxamer188-*b*-PCL. The thermo-stability of poloxamer188 -*b*-PCL was carried out with a thermal gravimetric analyzer (TGA), and cytotoxicity was obtained using the CCK8 method. Cargo-free and curcumin (CUR)-loaded poloxamer188-*b*-PCL NPs were fabricated via the solvent evaporation method. The morphology, particle size distribution, and stability of cargo-free NPs were studied with a scanning electron microscope (SEM) and laser particle analyzer. The *in vitro* radioprotection activity of CUR-loaded NPs was performed. FTIR, Raman, and ^1^H NMR spectra confirmed that poloxamer188-*b*-PCL was obtained. TGA curves suggested poloxamer188-*b*-PCL had better thermo-stability than original poloxamer188. Cell tests suggested that the cargo-free NPs had no cytotoxicity. SEM image showed that the cargo-free NPs were spherical with a diameter of 100 nm. Free radical scavenging experiments proved that CUR-loaded NPs had better antioxidant activity than CUR solutions. CUR-loaded NPs could be detected in all tissues, including liver, kidneys and lung. In summary, this work demonstrated a feasibility of developing an injective formulation of CUR and provided a protection agent in caner radiotherapy.

## Introduction

Curcumin (CUR), a natural chemical, is first extracted from the rhizomes of zingiberaceae or araceae plants (Zhao et al., 2015). CUR is an infrequent diketone pigment which is distributed in plants. Because of the low toxicity, CUR is applied as a colorant in the field of foods (Mishra and Daswal, 2007; Epstein et al., 2010; Lüer et al., 2014), which is approved by the World Health Organization (WHO), the Food and Drug Administration (FDA), and many countries. In recent years, CUR is been one of the top-selling natural edible pigments. Furthermore, CUR has broad clinical application, i.e., antioxidant activity (Esatbeyoglu et al., 2015; Llano et al., 2019; Wang et al., 2019), anti-tumor (Ohtsu et al., 2002; Kunnumakkara et al., 2007; Yan et al., 2017), anti-inflammatory (Aggarwal and Harikumar, 2009; Fadus et al., 2017; Hussain et al., 2017), and anti-hyperlipidemia (Huang et al., 2018; Panahi et al., 2018; Wang et al., 2018), and so on. In particular, bioactivities of curcumin as an effective chemopreventive agent, chemo-/radio-sensitizer for tumor cells, and chemo-/radio-protector for normal organs, are of extraordinary research interests in the literature (Farhood et al., 2018).

However, in their clinical applications, CUR molecules show certain drawbacks, e.g., low solubility (Lim et al., 2018; Peng et al., 2018), poor stability (Kharat et al., 2017; Luo et al., 2017), low absorptivity (Gopi et al., 2017), and short half-life (Hussain et al., 2017). These weaknesses lead to the low bioavailability of CUR and limit its further applications. It is reported that CUR can be detected in the body when the oral dosage is up to 10–12 g. Administrating 10 mg/kg of CUR by intravenous injection, the peak plasma concentration of a rat is only 0.36 μg/ml. After 15 min of gavage (1.0 g/kg CUR), the concentration of CUR in rat plasma is only 0.13 μg/ml, and 1.0 h later, the peak plasma concentration is only 0.22 μg/ml (Akinyemi et al., 2017). Six hours later, CUR cannot be detected in rat blood. After gavage, 90% CUR is found in the stomach and small intestine, and few CUR molecules can be detected in the blood, liver, and kidneys. Therefore, improving the stability and bio-availability of CUR will be important for future directions.

It has been reported that polymeric nanoparticles (NPs) can be used to improve the water solubility and stability of hydrophobic drugs (Levard et al., 2012; Krull et al., 2017). Therefore, CUR-loaded polymer NPs can prevent these molecules from being oxidized and improve their stability as well as their bioavailability. For biological applications, the matrix of NPs should have good biodegradability, biocompatibility, and no cytotoxicity. Poloxamer188, a non-ionic surfactant, is the block copolymer of polyoxyethylene and polypropylene oxide (Armstrong et al., 1995). Due to its excellent biocompatibility and low bio-toxicity, poloxamer188 is widely used in pharmaceuticals to improve the solubility of hydrophobic molecules (Ofokansi et al., 2012), enhance the stability of model drugs (Huang et al., 2008), and accelerate the absorbance of bioactive components. Furthermore, Poloxamer188 has a terminal hydroxyl group, which can be modified for further application. Caprolactone (CL) is a non-toxic chemical intermediate, which is usually used as monomers to fabricate Polycaprolactone (PCL) which is a high performance polymer. Due to its excellent bio-compatibility, PCL can be used as the material support growth of cells (John and Qi, 2008). In addition, good bio-degradability allows PCL to be completely degraded within 12 months in the natural environment.

Poloxamer 188 (Pluronic F68, F68) employed as a surfactant, can cause sensitization of multiple drug resistance (MRD) tumors to various anticancer agents and enhance drug transport across blood brain and intestinal barriers. Poloxamer188 blended into PCL could affect the microspheres' morphology and control drug release (Ma and Song, 2007). In this study, a novel copolymer was prepared via a ring-opening polymerization with poloxamer188 and ε-CL as monomers. Hydrogen nuclear magnetic spectra (^1^H NMR), Fourier transform infrared spectroscopy (FTIR), Raman spectra were used to confirm the obtained poloxamer188-*b*-PCL. CUR was selected as a model drug and loaded into the poloxamer188-*b*-PCL NPs to improve the stability of CUR. The morphology, size distribution, and stability of obtained NPs were studied. The *in vitro* free-radical-scavenging ability and antioxidant activity were also investigated to preliminarily evaluate its radioprotection ability.

## Experimental

### Materials and Methods

Poloxamer188 was the product of BASF (Germany). ε-CL, Tin(II) 2-ethylhexanoate, and CUR were bought from Energy chemical (Shanghai, China). Tetrahydrofuran (THF, CP) was obtained from Sinopharm Chemical Reagent Co., Ltd. 2, 2′-azino-bis(3-ethylbenzothiazoline-6-sulfonic acid) (ABTS) and 1,1-diphenyl-2- picrylhydrazyl (DPPH) were the products of Energy chemical (Shanghai, China). Trichloroacetic acid, potassium ferricyanide, and ferric trichloride were obtained from Aladdin Industrial Corporation (Shanghai, China). Other organic reagents were bought from Tianjin Beichen chemical reagent Co., Ltd (Tianjin, China), and used as received without any further purification.

### Synthesis of Poloxamer188-*b*-PCL

**Figure 1:**
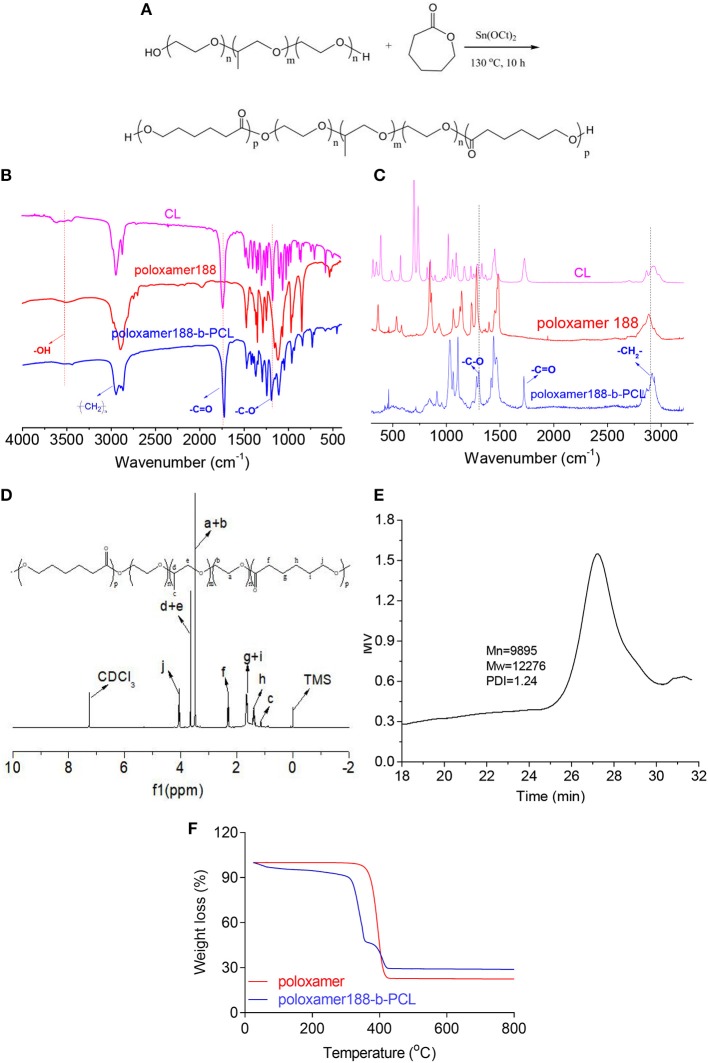
Synthetic process of poloxamer188-*b*-PCL polymers **(A)**. The block polymers were synthesized via the ring-opening polymerization with poloxamer188 and ε-CL as starting materials; FTIR spectra of ε-CL, poloxamer188, and poloxamer188-*b*-PCL polymers **(B)**. The absorption peak of –C=O group was found in the spectrum of final polymer, which confirmed the obtaining of the poloxamer188-*b*-PCL polymers; Raman spectra of ε-CL, poloxamer188, and poloxamer188-*b*-PCL polymers **(C)**; ^1^H NMR spectrum of poloxamer188-*b*-PCL polymers **(D)**. The ^1^H NMR spectrum also confirmed the obtaining of poloxamer188-*b*-PCL polymers; GPC curve of poloxamer188-*b*-PCL polymers **(E)**. The Mw of obtained polymer was 12,276 D; TGA curves of poloxamer188 and poloxamer188-*b*-PCL polymers **(F)**. TGA curves indicated that the poloxamer188-*b*-PCL polymers improved the thermo-stability of poloxamer188.

Poloxamer188-*b*-PCL was prepared via a ring-opening polymerization with Poloxamer188 and ε-PCL as monomers (as shown in Figure 1A). 1.60 g of poloxamer188 and 200 μl of Tin(II) 2-ethylhexanoate were dissolved into 3.20 g of ε-CL. Next, air in the reaction tube was removed by a vacuum pump, and then the tube was sealed up in nitrogen atmosphere. After being reacted at 130°C for 10.0 h, the crude poloxamer188-*b*-PCL was synthesized. Then 1.0 ml methylene chloride was used to dissolve the crude products, and the refined polymers were obtained by dropwise adding the above mentioned solution into 50 ml ice-cooled methyl alcohol. After filtering, refined poloxamer188-*b*-PCL dried at 40°C for 24 h.

### Characterization on Copolymers

The FTIR spectra of poloxamer188, ε-CL, and PCL were obtained from an FTS-135 (BIO-RAD, USA) spectrometer, scanning from 4,000 to 500 cm^−1^. KBr pellets were prepared under a hydraulic pressure of 400.0 kg at the KBr and specimen ratio of 10:1 (m/m). Meanwhile, Raman spectra were also used to study the structure of final copolymers. All spectra were recorded on a Metage OPAL Portable Raman System (ProRoman L-785, EVWAVE Optronics. lnc), covering a range of 3,200–3250 cm^−1^ at a spectral resolution of 4 cm^−1^, and each sample was scanned for 10 times.

^1^H NMR spectrum was used to analyze the structure of the obtained copolymers. ^1^HNMR spectra were recorded on a Bruker 400 MH_Z_ nuclear magnetic resonance instrument with CDCl_3_.

Mettler-Toledo TGA/DSC-2 (Switzerland) was sued to study the thermo-stability of poloxamer188 and poloxamer188-*b*-PCL. About 5.0 mg of samples were loaded into ceramic pans (50 μl), and their TGA curves were recorded under the atmosphere of high purity nitrogen (flow rate of 20 ml/min) heating from 25 to 500°C at the rate of 10°C/min.

The molecular weight of poloxamer188-*b*-PCL was measured with Waters 1515 GPC (Waters Company, USA). THF was selected as the mobile phase, and the flow rate was set as 1.0 ml/min. Polystyrene with the molecular weights of 100–500,000 were used as reference substances.

### Fabrication of Poloxamer188-*b*-PCL NPs

Poloxamer188-*b*-PCL NPs were fabricated by a solvent evaporation method (Alshamsan, 2014). One hundred milligrams of poloxamer188-*b*-PCL was dissolved into 3.0 ml THF. Under magnetic stirring (350 rpm), the polymer/THF solution was slowly added into 100 ml deionized water. Then, the solution was stirred for another 12 h. After removing the residual THF, the poloxamer188-*b*-PCL NPs were fabricated. The CUR-loaded NPs were fabricated with the same method. 10.0 mg CUR and 100 mg poloxamer188-*b*-PCL were dissolved into 3.0 ml THF, and the other steps were the same as described above.

### Stability of Cargo-Free NPs

Six test tubes each with 4.0 ml cargo-free NPs solutions were incubated in a 80°C water bath. At the time points of 0 min, 10 min, 20 min, 30 min, 40 min, and 60 min, one tube was fetched to measure the size distribution of the cargo-free NPs. 2.0 ml NPs solution was loaded into a cuvette, and the size distribution was measured with a Malvern Zatasizer (Nano-ZS90, UK) under room temperature. Each sample was measured for three times.

10% of Bovine Serum Albumin (BSA) was added into a cargo-free NPs solution to study their colloidal stability (Zuo et al., 2015). The NPs solution without BSA was used as a control. At the time points of 0, 2, 4, 6, 8, 10, 12, and 24 h, the hydrodynamic diameters and PDI of NPs solutions were measured with a Malvern Zatasizer (Nano-ZS90, UK).

### Cytotoxicity

The cytotoxicity of cargo-free poloxamer188-*b*-PCL NPs was carried out with a CCK8 method. KYSE520 cells were seeded in 96-well plates at a density of 8.0 × 10^3^ cells/ml. These cells were incubated at 37°C for 48 h with 10.0 μl of cargo-free NPs solutions with the concentrations of 30.0, 60.0, 250, and 1,000 μg/ml, respectively. Before harvest, 10.0 μl CCK8 solutions were added into the wells. After additional 4 h incubation at 37°C, the absorbance was measured at 450 nm using a microplate reader (Bio-Rad Model 680, UK).

### Cells Uptake

KYSE520 cells were seeded in 6-well plates at the density of 10, 000 cells per well. The culture medium was kept at 37°C and maintained in a humidified atmosphere containing 95% air and 5% CO_2_. Forty-eight hours later, 100 μg/ml CUR/DMSO solution and CUR-NPs suspension (containing 100 μg/ml CUR) were added into the culture medium. The cells were incubated for another 8 h, and then the cells were washed with 1.0 ml phosphate buffer solution (10 mM, pH 7.4) three times to remove the free CUR and CUR-NPs. The fluorescence intensities of cells were observed with a laser scanning confocal microscope (LSCM, Leica AF 6500, Germany).

### *In vitro* Antioxidant Activity

#### ABTS Free Radical (ABTS·^+^) Scavenging Experiments (Li et al., 2017; Rashed et al., 2018)

38.4 mg of ABTS was dissolved into 10 ml deionized water to prepare the ABTS base solution (7 mM). K2S2O8/H2O solution (4.9 mM) was used as K2S2O8 base solution. Both solutions were mixed together and stayed for 12–16 h to be used as the ABTS·^+^ stock solution. Before usage, the absorbance of stock solution was diluted to A734 = 0.7± 0.02 with phosphate buffer (PBS, pH = 7.4), which was used as operating fluid.

The test fluids were CUR-loaded NPs solutions and CUR/alcohol solutions with the CUR concentrations of 0, 1, 5, 10, 20, 25, and 50 μg/L, respectively. One hundred and forty microliters of test fluids were added into 4.0 ml ABTS·^+^ operating fluid (A_734_ = 0.7 ± 0.02). After a 6 h reaction in a 30^o^C water bath, the absorbance of the above mentioned solutions was measured with a UV-2700 ultraviolet spectrophotometer (734 nm). PBS (pH = 7.4) was selected as the blank control. The clearance rate of ABTS·^+^ was calculated following Equation (1):

(1) $$\begin{array}{r} C_{ABTS^{\cdot +}}=\frac{A_{c}-A_{s}}{A_{c}}\times 100 \end{array}$$

Where: *C*_*ABTS*·+_ was the clearance of ABTS·^+^; *A*_*s*_ and *A*_*c*_ were absorbance of samples and blank controls, respectively. When the absorbance of ABTS·^+^ clearance was 50%, the sample concentrations were recorded as SC_50_.

#### DPPH Free Radical Scavenging Experiments (Ju et al., 2011; Ullah et al., 2017)

1.9716 g of DPPH was dissolved into 50 ml alcohol to be used as a stock solution, which was kept in a dark place under room temperature. CUR-loaded NPs solutions and CUR/alcohol solutions with concentrations of 0, 1, 5, 10, 20, 25, and 50 μg/L were selected as test solutions. 2.0 ml of the test solution was added into the isopycnic DPPH solution, and the mixture was kept in a dark place for 30 min. The absorbance of the above mentioned solution was then measured with a UV-2700 ultraviolet spectrophotometer at 517 nm. The clearance rate of ABTS·^+^ was calculated following Equation (2):

(2) $$\begin{array}{r} C_{\text{DPPH}}=\frac{A_{c}-A_{s}}{A_{c}}\times 100 \end{array}$$

Where: *C*_*DPPH*_ was the clearance of ABTS·^+^; *A*_*s*_ and *A*_*c*_ were absorbance of samples and blank controls, respectively. When the absorbance of clearance of DPPH was 50%, the sample concentrations were recorded as SC_50_.

#### Reducing Power (Shabbir et al., 2013)

The mixture of 2.0 ml test solution, 2.5 ml potassium ferricyanide solution and 2.5 ml PBS (pH 6.82) was incubated in a 50°C water-bath for 20 min. When the mixture was cooled in an ice-water bath, another 2.5 ml trichloroacetic acid solution (10 w/w%) was added into the above mentioned mixture. After centrifugation (3,000 r/min, 10 min), 2.5 ml supernate was added into the mixture of 2.5 ml deionized water and 0.5 ml ferric trichloride solution (0.1 w/w%). Ten minutes later, the absorbance of the mixture was measured with a UV-2700 ultraviolet spectrophotometer at 710 nm.

### *In vivo* Distribution of Poloxamer188-*b*-PCL NPs

In the animal experiment, rhodamine B (RhB) was used as a fluorescence probe. RhB-loaded Poloxamer188-*b*-PCL NPs were prepared following the method which was described above (section Fabrication of Poloxamer188-*b*-PCL NPs). C57 rats were treated with 200 μl RhB-loaded Poloxamer188-*b*-PCL NPs (10 mg/ml) by intraperitoneal injection. At proper intervals, the rats were sacrificed and their viscera (i.e., heart, liver, spleen, lungs, and kidneys) removed. The fluorescence intensity of all the viscera was examined using the CRI Maestro *in vivo* imaging system (CRI Corporation, Woburn, MA, USA) at 523 nm.

All of rats were housed in individual cages in a controlled environment with free access to food and water. The experiment was carried out in accordance with the People's Republic of China national standards (GB/T16886.6-1997).

## Results and Discussion

### Characterization on Poloxamer188-*b*-PCL

FTIR spectra of the obtained polymer were shown in Figure 1B. In the spectrum of ε-CL, the absorption peak observed at 1742.9 cm^−1^ was due to the stretching vibration of carbonyl groups (Yin et al., 2017). By contrast, this peak was not observed in the spectrum of poloxamer188. However, such peak was found in final polymers. Further, the peak at 3,515.5 cm^−1^ could be attributed to the stretching vibration of –OH (Barka et al., 2013), which was observed in the spectra of poloxamer188-*b*-PCL. These data indicated that the esterification reaction was performed between poloxamer188 and ε-CL. Raman spectra gave the similar results (as shown in Figure 1C). The diffraction maximum at 1,720 cm^−1^ (-C=O) was observed in the spectra of ε-CL and final polymers. Besides, another diffraction maximum at 1,250 cm^−1^ was assigned to the vibration of -C-O groups. Therefore, it could be speculated that new ester bonds generated in the final polymers.

The ^1^H NMR spectrum of final polymers is shown in Figure 1D. It can be seen that bands assigning to the protons of PCL chains were observed at 2.3 ppm (-CO-**CH**_**2**_-, f), 1.6 ppm (-**CH**_**2**_-, g+i), 1.4 ppm (-**CH**_**2**_-, h), and 4.0 ppm (-O-**CH**_**2**_-, j), respectively (Ali et al., 2017). The bands of H atoms assigned to poloxamer188 were observed at 1.0 ppm (-**CH**_**3**_, c), 3.7 ppm (-O-C**H**_**2**_-C**H**-, d+e), and 3.5 ppm (-C**H**_**2**_-C**H**_**2**_-O-, a+b), respectively (Jacobsson, 2005). Figure 1E provides the GPC results of poloxamer188-*b*-PCL. It can be seen that the GPC curve was a single summit structure which was symmetrically distributed. The PDI (M_w_/M_n_) of poloxamer188-*b*-PCL was 1.24, which suggested that the molecular weight distribution was narrow. In conclusion, these studies confirmed that poloxamer188-*b*-PCL was obtained.

The thermo-stability of poloxamer188-*b*-PCL was studied and the TGA results are shown in Figure 1F. The initial decomposition temperature of poloxamer188 was observed at 340°C with a mass loss of 74%, which could be attributed to the breaking of ether bonds and –OH groups. By contrast, two decomposition events were found in the TGA curve of poloxamer188-*b*-PCL. The first event was observed at 300–350°C with a mass loss of 50%, which was assigned to the decomposition of poloxamer188 chains (Abdelrazek et al., 2016). The event at 370–410°C (mass loss of 15%) was due to the thermal rupture of PCL chains (Nadal et al., 2016). The differences between both curves indicated that poloxamer188-*b*-PCL had better thermo-stability than original poloxamer188.

### Stability of Poloxamer188-*b*-PCL NPs

**Figure 2:**
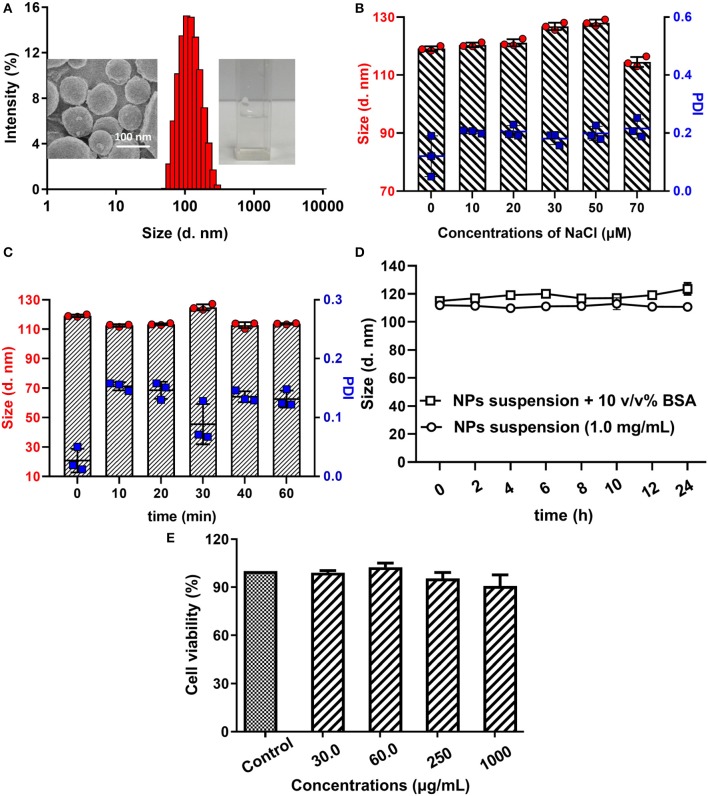
Size distribution of poloxamer188-*b*-PCL NPs **(A)**, SEM image of cargo-free NPs (insert, left), and poloxamer188-*b*-PCL NPs solution (insert, right). The average diameter of the obtained NPs was 119.9 ± 0.5 nm with a PDI of 0.03 ± 0.02; the different stability of cargo-free NPs in various NaCl solution **(B)**. The cargo-free NPs showed an excellent stability in NaCl solutions; the thermo-stability of cargo-free NPs **(C)**. The size distribution of poloxamer188-*b*-PCL NPs in BSA solution **(D)**. After 60 min incubation under 80^o^C, the cargo-free NPs had good stability; the stability of cargo-free NPs in 10% BSA solution; Cytotoxicity of the cargo-free NPs **(E)**. Cell results indicated that the poloxamer188-*b*-PCL NPs did not show obvious cytotoxicity.

In ultrapure water, the amphipathic polymer poloxamer188-*b*-PCL could self-assemble into NPs with hydrophobic cores and hydrophilic shells. The obtained cargo-free NPs showed a diameter of 119.9 ± 0.5 nm with a PDI of 0.03 ± 0.02 (Figure 2A), which suggested that the particle size distribution of obtained NPs was narrow and well-proportioned. Furthermore, it can be seen from SEM image (Figure 2A insert, left**)** that the obtained NPs were spherical with a diameter of about 100 nm, which is consistent with the results obtained from the laser particle analyzer. Similarly, the concentrations of NaCl did not influence the size distribution of cargo-free NPs (as shown in Figure 2B).

Heating could increase the energy of NPs, and conglomeration would happen among NPs. The thermo-stability of cargo-free NPs was studied in a 80°C water-bath, and results were shown in Figure 2C. It could be seen that the cargo-free NPs showed excellent thermo-stability. During the water-bath (80°C), the NPs' diameters were fluctuated between 110 and 120 nm, and the PDIs were all in the range of 0.02–0.2. These data indicate that the obtained NPs had a good thermodynamic stability.

After adsorption by protein, it was difficult for the NPs to be delivered to the targets, and the drug-loaded NPs would not be effective. Therefore, the NPs should have protein resistance. As shown in Figure 2D, although the particle size distribution of the NPs solution with 10% BSA was slightly bigger than that of the original NPs solution, their diameters still fluctuated between 110 and 120 nm. This phenomenon illustrates that the poloxamer188-*b*-PCL NPs could not be adsorbed by BSA, and had good protein resistance.

Furthermore, the biomaterial should be non-toxic. The cytotoxicity of poloxamer188-*b*-PCL NPs was reflected by cell viability. In Figure 2E, in incubation with the NPs solutions (30.0, 60.0, 250, and 1,000 μg/ml, respectively), the viability of KYSE520 cells were over 90%. The high viability of all cells indicates that the cargo-free poloxamer188-*b*-PCL NPs had no cytotoxicity, and could be used as a biomaterial.

### *In vitro* Antioxidant Activity

**Figure 3:**
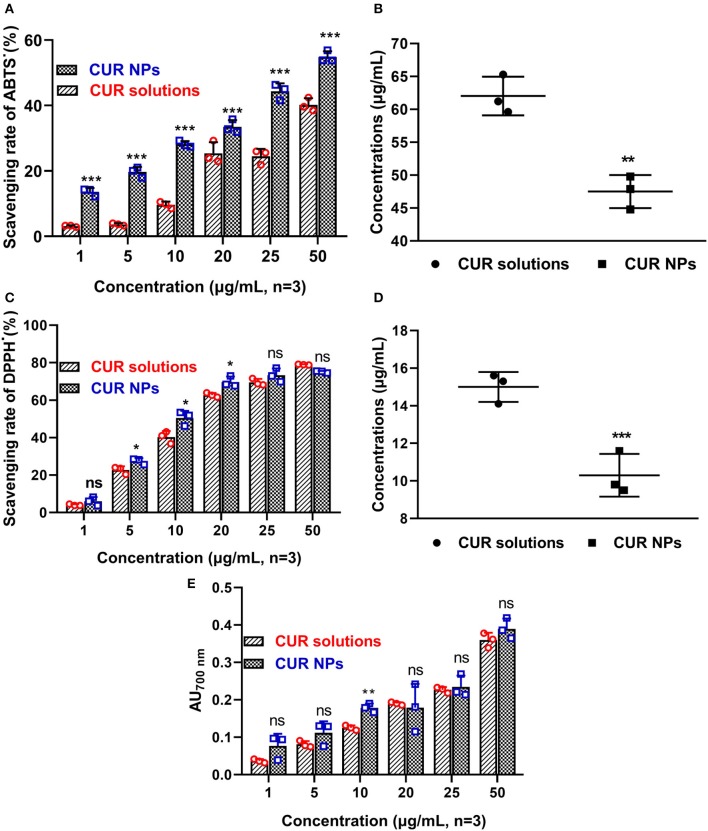
ABTS·^+^ scavenging experiments of original CUR and CUR-loaded poloxamer188-*b*-PCL NPs **(A)**. The CUR-loaded NPs had better ABTS·^+^ scavenging rate than that of original CUR powder; The SC_50_ of original CUR and CUR-loaded poloxamer188-*b*-PCL NPs **(B)**; DPPH· scavenging experiments of original CUR and CUR-loaded poloxamer188-*b*-PCL NPs **(C)**. The CUR-loaded NPs had better DPPH^∙^ scavenging rate than that of original CUR powder; The SC_50_ of original CUR and CUR-loaded poloxamer188-*b*-PCL NPs **(D)**; the reducing power of original CUR and CUR-loaded poloxamer188-*b*-PCL NPs **(E)**. *p < 0.05; **p < 0.01; ***p < 0.001.

After 24 h, the ABTS^.+^ scavenging experiments were carried out, and their scavenging rates can be seen in Figure 3A. In different concentrations, CUR-loaded NPs gave higher ABTS^.+^ scavenging rates. After a *t*-test analysis, significant differences were found between CUR-loaded NPs and CUR/alcohol solutions (^***^P < 0.001). As shown in Figure 3B, the CS50 of CUR/alcohol solutions and CUR-loaded NPs were 62.3 ± 2.9 and 47.5 ± 2.6 mg/ml, respectively (^**^P < 0.01). Similarly, CUR-loaded NPs exhibited excellent DPPH scavenging effects (Figure 2C). Twenty-four hours later, most CUR-loaded NPs solutions had higher DPPH scavenging rates than those of CUR/alcohol solutions (^*^P < 0.05) (as shown in Figure 3C). The CS50 of CUR-loaded NPs and CUR/alcohol solutions were 15.0 ± 0.8 and 10.3 ± 1.1 mg/ml, respectively (^***^P < 0.001) (as shown in Figure 3D). These differences could be attributed to the various stabilities of CUR. In CUR/alcohol solutions, CUR molecules were easily oxidized by oxygen, while the CUR-loaded NPs were difficult to destroy. Therefore, the new prepared CUR/alcohol solutions had better antioxidant activities than those of CUR-loaded NPs (Figure 1S). However, 24 h later, with the oxidization of CUR/alcohol solutions, their antioxidant activities decreased.

It was reported that the antioxidant activities of substances were proportional to their reducing power. The reducing materials provided H atoms with the ability to break the free radial chains, which generated antioxidant activities. In this study, the H atoms from CUR reduced Fe^3+^ into Fe^2+^, which prevented the generation of peroxide. This process is a Prussian blue reaction, and the products had a maximum absorbency of 700 nm. The higher absorbency suggested the sample had better reducing power. The reducing powers of CUR-loaded NPs and CUR/alcohol solutions are shown in Figure 3E. Most CUR molecules were encapsulated in NPs, and newly prepared CUR/alcohol solutions had better reducing powers than CUR-loaded NPs (Figure 2S). However, 24 h later, both materials had similar reducing powers. In general, CUR-loaded NPs had better *in vitro* antioxidant activity than that of CUR/alcohol solutions.

The information on curcumin suggests that the radioprotective effect might be mainly due to its ability to reduce oxidative stress after exposure ionizing radiation. Therefore, CUR-loaded NPs will have better clinical efficacy than a curcumin drug during radiotherapy of cancer.

### Cell Uptake

**Figure 4:**
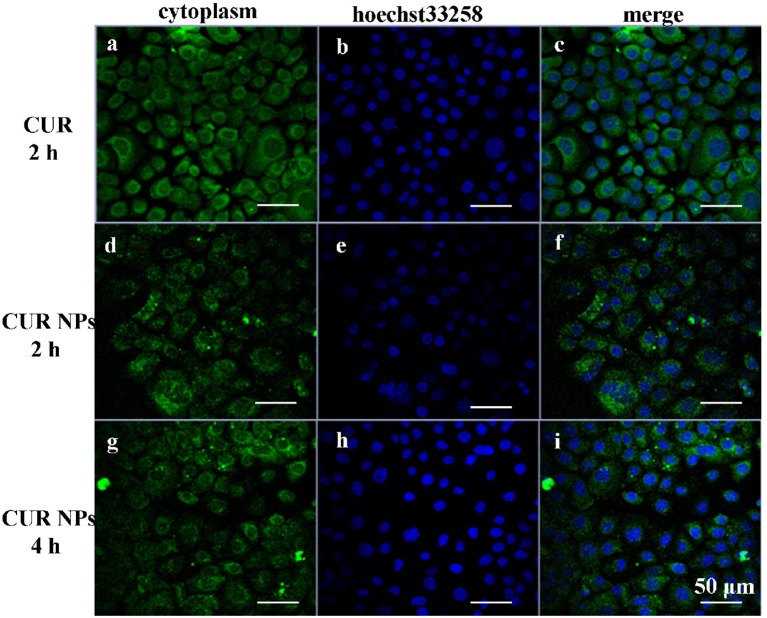
Fluorescence microscopy of KYSE520 cells incubated with free CUR and CUR-loaded NPs at equivalent CUR concentrations. It could be seen that the CUR-loaded NPs could be ingested by KYSE520 cells. **(a)** The cytoplasm of KYSE520 cells after 2 h of treating with CUR; **(b)** The cell nucleus of KYSE520 cells after 2 h of treating with hoechst33258; **(c)** The merge image of **(a,b)**; **(d)** The cytoplasm of KYSE520 cells after 2 h of treating with CUR NPs; **(e)** The cell nucleus of KYSE520 cells after 2 h of treating with hoechst33258; **(f)** The merge image of **(d,e)**; **(g)** The cytoplasm of KYSE520 cells after 4 h of treating with CUR NPs; **(h)** The cell nucleus of KYSE520 cells after 4 h of treating with hoechst33258; **(i)** The merge image of **(g,h)**.

Usually, cells uptake CUR molecules via free diffusion, while CUR-loaded NPs enter cells via endocytosis. Therefore, CUR-loaded NPs provided lower fluorescence intensity than that of CUR molecules. Figure 4 shows the fluorescence photographs of KYSE520 cells incubated with CUR solutions and CUR-loaded NPs, respectively. At 2 h, more CUR molecules were up-taken by cells. Therefore, KYSE520 cells treated with CUR molecules showed stronger fluorescence intensity than those treated with CUR-loaded NPs. However, with time, more CUR-loaded NPs entered into cells and more CUR molecules were decomposed by cells, and both batches of cells had a similar fluorescence intensity. These phenomena illustrate that the CUR-loaded NPs could be endocytosed by Cells, and had better stability than CUR solutions.

### Bio-Distribution of RhB-Loaded Poloxamer188-*b*-PCL NPs

**Figure 5:**
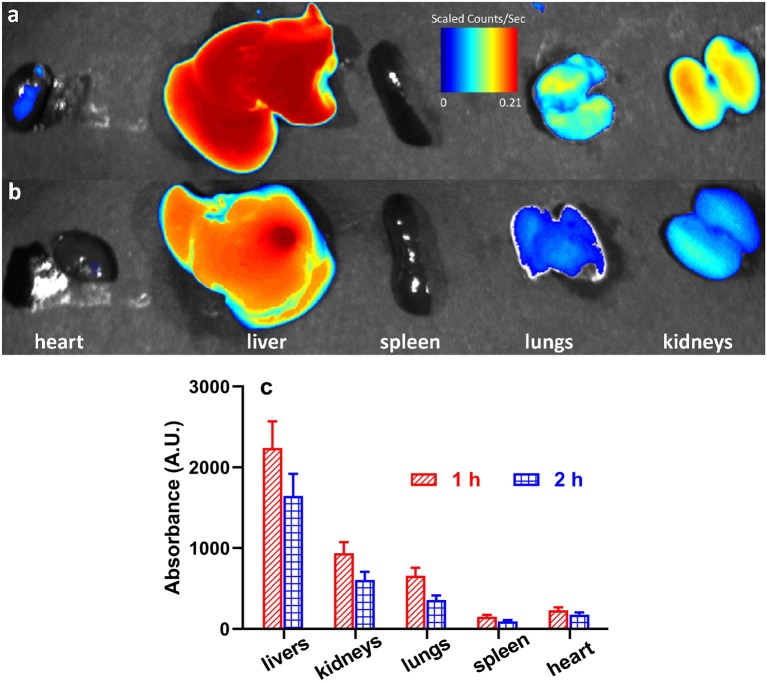
Representative fluorescent images of rat's viscera (i.e., heart, liver, spleen, lungs, and kidneys) after 1 h **(a)** and 2 h **(b)** of intraperitoneal injection of RhB-loaded NPs; the fluorescent intensity of heart, liver, spleen, lungs, and kidneys **(c)**.

The *in vivo* distribution of the Poloxamer188-*b*-PCL NPs was carried out with RhB as a fluorescence probe. Figures 5a,b displays the RhB levels in various viscera, i.e., heart, liver, spleen, lungs, and kidneys. After intraperitoneal injection, the RhB-loaded NPs could distribute fast in all tissues. The RhB-loaded NPs were mainly distributed in liver, lungs, and kidneys (Figure 5a). The highest fluorescence intensity was found in liver, which was over 2000 A.U., and the spleen showed the lowest intensity at 135 A.U. (as shown in Figure 5c). Similar results were observed after 2 h of injection (Figure 5b), but all fluorescence intensity decreased. The result revealed NPs selective distribution in different tissues. The presence of NPs in the kidney and lung is of special interest because protection of these tissues from radiation damage is key to recovery and host survival during caner radiotherapy.

## Conclusions

A novel block polymer of poloxamer188-*b*-PCL was synthesized via the ring-opening polymerization. ^1^H NMR, Raman, and FTIR spectra confirmed that poloxamer188-*b*-PCL polymers were obtained. The GPC curve showed that the Mw of poloxamer188-*b*-PCL polymers was 12,000 D. The poloxamer188-*b*-PCL NPs were fabricated using a solvent-evaporation method. The obtained NPs showed an average diameter of about 100 nm. The cargo-free NPs showed a high stability in different NaCl solutions and temperature conditions. Cell tests indicated that the poloxamer188-*b*-PCL NPs did not have obvious cytotoxicity. As a potential radioprotection agent, CUR-loaded poloxamer188-*b*-PCL NPs improved the *in vitro* antioxidant activity of the crude CUR powder. Importantly, the CUR-loaded poloxamer188-*b*-PCL NPs could be ingested by KYSE520 cells. The animal experiment showed that RhB-loaded NPs could bio-distribute into the liver, kidney, and lung. Therefore, the poloxamer188-*b*-PCL NPs improved the stability and antioxidant activity of CUR. Furthermore, such CUR loaded NPs provided a potential protector against damage from radiotherapy cancer treatment.

## Data Availability Statement

All datasets generated for this study are included in the article/Supplementary Material.

## Ethics Statement

The animal study was reviewed and approved by Institute of Radiation Medicine, Chinese Academy of Medical Science & Peking Union Medical College.

## Author Contributions

YS and XL designed experiments and analyzed experimental results and wrote the manuscript. SY, WL, SL, ML, SC, YW, and MC carried out *in vitro* experiments. XL carried out *in vivo* experiments.

### Conflict of Interest

The authors declare that the research was conducted in the absence of any commercial or financial relationships that could be construed as a potential conflict of interest.
