# Supplementary material for: Preparation of Poloxamer188-b-PCL and Study on in vitro Radioprotection Activity of Curcumin-Loaded Nanoparticles
Source: Front Chem. 2020 Apr 15;8:212. doi: 10.3389/fchem.2020.00212 (PMC7174741; doi:10.3389/fchem.2020.00212)
Supplement: Supplementary file 1 [file Data_Sheet_1.pdf]

Supplementary Material

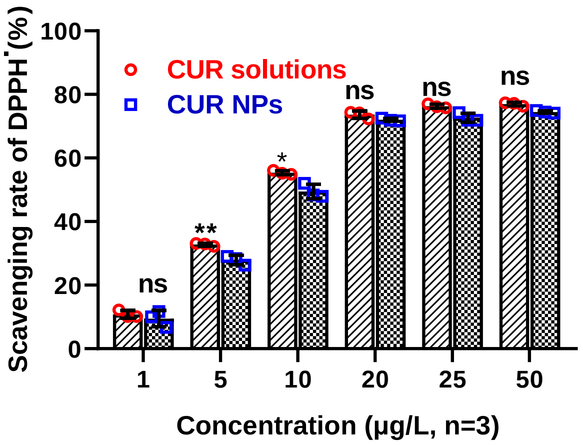

Figure 1S DPPH· scavenging rates of new prepared CUR/alcohol solutions and CUR-loaded NPs with the concentrations of 1.0 mg/mL, 5.0 mg/mL, 10 mg/mL, 20 mg/mL, 25 mg/mL, and 50 mg/mL, respectively. Most of CUR molecules were encapsulated in the hydrophobic cores of poloxamer188-b-PCL NPs. Therefore, comparing with CUR/alcohol solutions, the new prepared CUR-loaded NPs had less DPPH· scavenging rates.

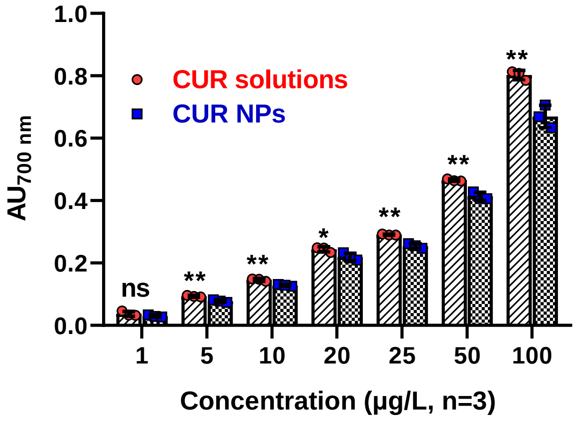

Figure 2S Reducing powers of new prepared CUR/alcohol solutions and CUR-loaded NPs. New prepared CUR/alcohol solutions showed better reducing powers than CUR-loaded NPs. However, with the oxidization of CUR molecules in their solutions and the CUR release from CUR-loaded NPs, CUR-loaded NPs gave the similar reducing powers as CUR/alcohol solutions (**Fig. 3e**).
